# Prediction of Chemical Respiratory and Contact Sensitizers by OX40L Expression in Dendritic Cells Using a Novel 3D Coculture System

**DOI:** 10.3389/fimmu.2017.00929

**Published:** 2017-08-04

**Authors:** Izuru Mizoguchi, Mio Ohashi, Yukino Chiba, Hideaki Hasegawa, Mingli Xu, Toshiyuki Owaki, Takayuki Yoshimoto

**Affiliations:** ^1^Department of Immunoregulation, Institute of Medical Science, Tokyo Medical University, Tokyo, Japan

**Keywords:** animal testing alternatives, 3D coculture, respiratory sensitizers, skin sensitizers, dendritic cells, OX40L

## Abstract

The use of animal models in chemical safety testing will be significantly limited due to the recent introduction of the 3Rs principle of animal experimentation in research. Although several *in vitro* assays to predict the sensitizing potential of chemicals have been developed, these methods cannot distinguish chemical respiratory sensitizers and skin sensitizers. In the present study, we describe a novel *in vitro* assay that can discriminate respiratory sensitizers from chemical skin sensitizers by taking advantage of the fundamental difference between their modes of action, namely the development of the T helper 2 immune response, which is critically important for respiratory sensitization. First, we established a novel three-dimensional (3D) coculture system of human upper airway epithelium using a commercially available scaffold. It consists of human airway epithelial cell line BEAS-2B, immature dendritic cells (DCs) derived from human peripheral blood CD14^+^ monocytes, and human lung fibroblast cell line MRC-5. Respective cells were first cultured in individual scaffolds and subsequently assembled into a 3D multi-cell tissue model to more closely mimic the *in vivo* situation. Then, three typical chemicals that are known respiratory sensitizers (ortho-phthaldialdehyde, hexamethylene diisocyanate, and trimellitic anhydride) and skin sensitizers (oxazolone, formaldehyde, and dinitrochlorobenzene) were added individually to the 3D coculture system. Immunohistochemical analysis revealed that DCs do not migrate into other scaffolds under the experimental conditions. Therefore, the 3D structure was disassembled and real-time reverse transcriptase-PCR analysis was performed in individual scaffolds to analyze the expression levels of molecules critical for Th2 differentiation such as OX40 ligand (OX40L), interleukin (IL)-4, IL-10, IL-33, and thymic stromal lymphopoietin. Both sensitizers showed similarly augmented expression of DC maturation markers (e.g., CD86), but among these molecules, OX40L expression in DCs was most consistently and significantly enhanced by respiratory sensitizers as compared to that by skin sensitizers. Thus, we have established a 3D coculture system mimicking the airway upper epithelium that may be successfully applied to discriminate chemical respiratory sensitizers from skin sensitizers by measuring the critical molecule for Th2 differentiation, OX40L, in DCs.

## Introduction

There are mainly two types of allergic responses: skin sensitization and respiratory sensitization. The former is an allergic response in the skin following skin contact such as allergic contact dermatitis, and the latter is an allergic response in the airways caused by inhalation, mostly asthma. Determining the sensitization potential of a chemical is an important safety assessment process. Two traditional tests are accepted by the Organisation for Economic Co-operation and Development for assessment of chemical sensitization potential: the guinea pig maximization test (1) and the Buehler test (2). Currently, the murine local lymph node assay is the gold standard assay for evaluation of chemical sensitization potential (3). The local lymph node assay assesses the sensitization potential by monitoring the induced proliferative response of lymphocytes in the draining lymph nodes following chemical treatment. This assay has been extensively evaluated and validated, and the proliferative response has been shown to be highly correlated with the sensitization potency of the test chemicals (4). However, a worldwide movement is emerging, and the use of animal models in safety testing of chemicals will be significantly limited due to introduction of the 3Rs principle of refinement, replacement, and reduction of animal experimentation in research wherever possible (5). Therefore, several *in vitro* assays to predict the respiratory sensitizing potential of chemicals have been developed, the direct peptide reactivity assay (6), KeratinoSens (7), the human cell line activation test (8), and the interleukin (IL)-8 Luc assay (9). Accurate identification of skin or respiratory sensitizers is very important, because the adverse health effects are quite severe and long-lasting and the risk management systems for them are quite different (10). However, these alternative methods cannot distinguish chemical respiratory sensitizers and skin sensitizers (11).

The use of three-dimensional (3D) cell culture is favored over two-dimensional cell culture because 3D culture provides morphology, function, and cell–cell contact interactions that better resemble *in vivo* conditions and thus actual physiological situations. A 3D coculture system resembling the physiological situation of the human upper airway was recently reported (12). This system consists of epithelial cells, dendritic cells (DCs), and fibroblast cells, representing the physiological barrier, immune sensing, and extracellular matrix production, respectively. Initially, these cells were grown in individual scaffolds and then assembled into a 3D multi-cell tissue model. To date, however, no study has applied this 3D coculture system to assess the sensitizing potential of chemicals *in vitro*.

Accumulating evidence has revealed that respiratory and skin sensitizers induce different immune responses: predominantly T helper (Th) 2 responses versus Th1-oriented responses mixed with Th2 and Th17 responses (13–19). Discrimination between respiratory and skin sensitizers was achieved by the assessment of cytokine profiles in the local lymph node assay; the respiratory sensitizers induced greater expression of molecules critical for the induction of Th2 immune responses, such as IL-4 and IL-4 receptor (R) α, compared to the skin sensitizers (17–19). IL-4 is the predominant differentiating factor of Th2 cells from naive CD4^+^ T cells as well as an important effector cytokine produced by Th2 cells (20).

In the present study, we developed a novel *in vitro* assay using a 3D coculture system resembling human upper airway epithelium, which may discriminate respiratory sensitizers from chemical skin sensitizers by taking advantage of the fundamental differences between their modes of action, namely the development of Th2 immune responses, which is critically important for respiratory sensitization.

## Materials and Methods

### Cell Culture

Human upper airway epithelial cell line BEAS-2B (CRL-9609) (21) and human lung fibroblast cell line MRC-5 (CCL-171) (22) were purchased from the American Type Culture Collection (Manassas, VA, USA). Cells were cultured at 37°C under 5% CO_2_/95% air in Eagle’s minimum essential medium (MEM; Gibco, Grand Island, New York, NY, USA) containing 10% fetal calf serum and 100 µg/ml kanamycin (Meiji Seika, Tokyo, Japan). Human peripheral blood monocytes were cultured in RPMI 1640 medium (Sigma-Aldrich, St. Louis, MO, USA) containing 10% fetal calf serum and 100 µg/ml kanamycin.

### Reagents

Three chemical skin sensitizers, oxazolone (4-ethoxymethylene-2-phenyl-2-oxazolin-5-one; OXA, purity ≥90%, E0753), formaldehyde (FA, purity 36.5–38%, F8775), and 2,4-dinitrochlorobenzene (DNCB, purity 97%, 138630), and three respiratory sensitizers, ortho-phthaldialdehyde (OPA, purity ≥97%, P1378), hexamethylene-1,6-diisocyanate (HDI, purity ≥98%, 52650), and trimellitic anhydride (TMA, purity 97%, B4600), were purchased from Sigma-Aldrich (Table S1 in Supplementary Material). A magnetic bead conjugated with monoclonal antibody (mAb) against CD14 was purchased from Miltenyi Biotec (Bergisch Gladbach, Germany). mAbs for human CD14 (HCD14), CD11c (cllne 3.9), and CD11c (EP1347Y) were obtained from BioLegend (San Diego, CA, USA), eBioscience (La Jolla, CA, USA), and Abcam (Cambridge, UK), respectively. Alex Fluor 647 anti-rabbit IgG was purchased from Thermo Fisher Scientific (Waltham, MA, USA). Human recombinant granulocyte macrophage colony-stimulating factor (GM-CSF) was purchased from Miltenyi Biotec. Human recombinant IL-4 was kindly provided from Schering-Plough (currently, Merck, Kenilworth, NJ, USA).

### Human Monocytes

Fresh human peripheral blood was collected from healthy volunteers and mononuclear cells were immediately purified by using Lympholyte-H (Cedarlane, Burlington, ON, Canada) density gradient centrifugation. Monocytes were further purified from peripheral blood mononuclear cells by positive selection using an AutoMACS Pro with a magnetic bead conjugated with mAb against CD14 (Miltenyi Biotec). The purity was analyzed by flow cytometry with PE anti-CD14 using a FACS Canto II (BD Bioscience, San Jose, CA, USA) followed by analysis with FlowJo Software (Tree Star, Ashland, OR, USA), and was routinely more than 99%.

This study was approved by the institutional review board of Tokyo Medical University (no. 3323). Written informed consent was obtained from all participants in accordance with the Declaration of Helsinki.

### Preparation of Immature DCs

Human CD14^+^ monocytes (1 × 10^6^ cells/ml) were stimulated in a 24-well plate by GM-CSF (50 ng/ml) and IL-4 (10 ng/ml) for 6 days. The purity of resultant immature DCs was analyzed after staining with anti-CD11c and was routinely more than 99%.

### Preparation of the 3D Coculture System

**Figure 1:**
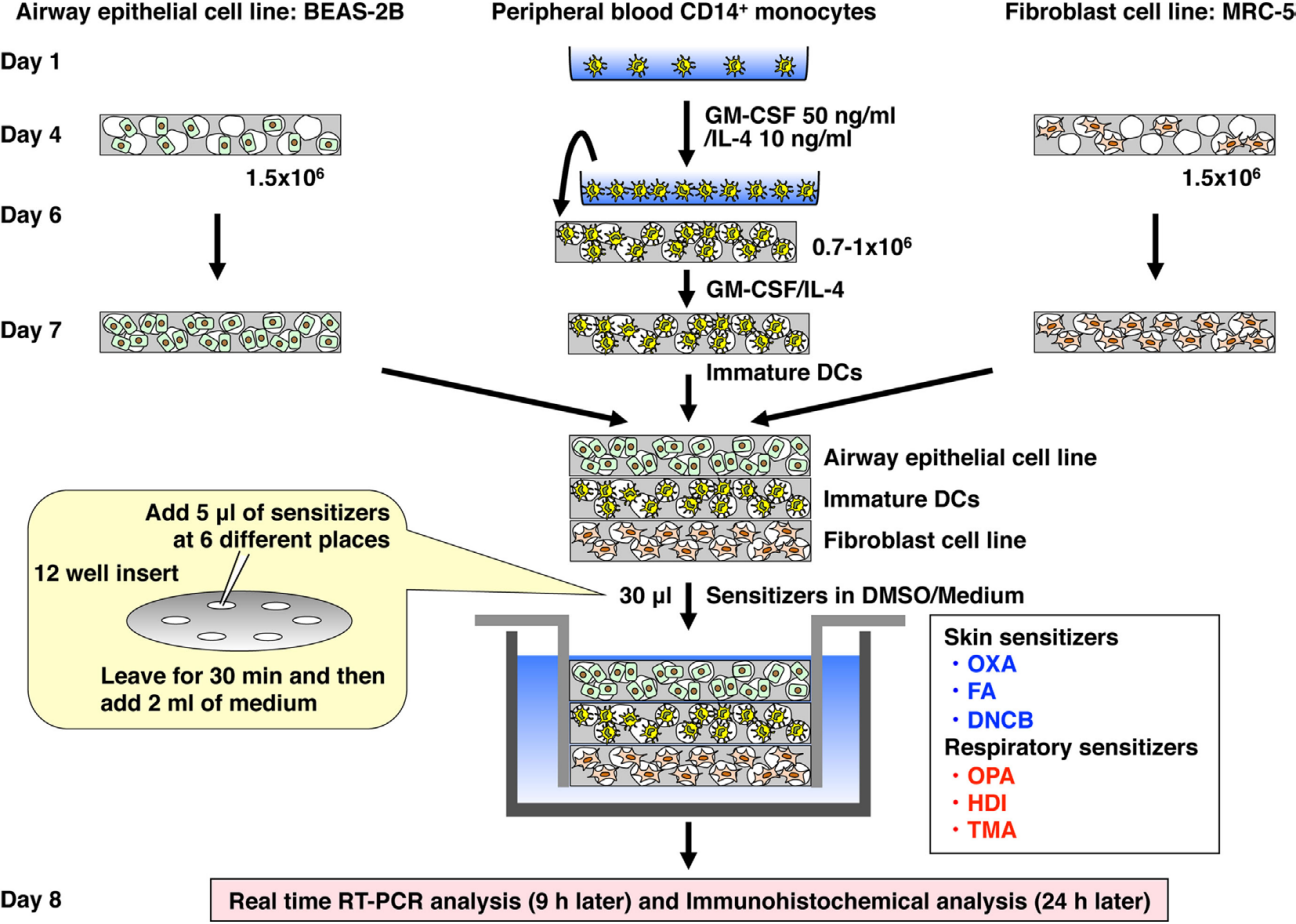
Establishment of a novel 3D coculture system. The system was established by using airway epithelial cell line BEAS-2B, peripheral blood mononuclear cell CD14^+^ monocyte-derived immature dendritic cells (DCs), and lung fibroblast cell line MRC-5 cultured initially in individual scaffolds and then assembled into the bottom of an insert well by piling the scaffolds.

The 3D coculture was prepared by using Alvetex scaffold 12-well inserts or 24-well plates, which were purchased from ReproCell (Glasgow, UK) (23). The scaffold was initially washed sequentially with ethanol, PBS, and medium according to the manufacture’s instructions. A 75-µl aliquot of cell suspension of BEAS-2B or MRC-5 cells (1.5 × 10^6^ cells) was gently seeded directly onto the center of the scaffold in the 12-well insert and left for 4 h to allow cell attachment. Then, medium was gently added on the scaffold and incubated for 3 days. A 50-µl aliquot of cell suspension of immature DCs (0.7–1.0 × 10^6^ cells) was gently seeded directly onto the center of the scaffold in the 24-well plate and left for 6 h. Then, an equal mixture of MEM and RPMI 1640 containing GM-CSF (50 ng/ml) and IL-4 (10 ng/ml) was gently added on the scaffold and incubated for 24 h. After incubation, the individual scaffolds were gently detached from the 12-well insert or 24-well plate and piled up in the order of MRC-5 cells (bottom), immature DCs (middle), and BEAS-2B cells (top), and attached to the bottom of a new sterile 12-well insert (Figure 1). Subsequently, the 12-well insert was placed in a 12-well plate, and MEM was gently added and incubated for another 4 h. Then, the medium was gently removed, and a 5-µl aliquot of a chemical sensitizer, which was initially dissolved in DMSO and then diluted with MEM, was gently added at six places on the top scaffold and left for 30 min followed by addition of MEM medium (2 ml). After stimulation for 9 h, the piled scaffold was disassembled, and RNA was extracted from the respective scaffolds for real-time RT-PCR analysis. After stimulation for 24 h, each scaffold was immersed in OCT embedding compound, snap-frozen in liquid nitrogen, and stored at −80°C until use.

### Immunohistochemical Analysis

Frozen samples were cut into10-μl sections by using a cryostat (Microm HM550, Thermo Fisher Scientific), fixed in 10% formalin, and stained with hematoxylin and eosin (HE). For DC staining with anti-CD11c (EP1347Y, Abcam), the fixed sections were incubated in a 0.05% Tween 20/citrate buffer (pH 6.0) solution at 60°C overnight to retrieve antigenicity according to the manufacture’s instructions. Then, the sections were stained with anti-CD11c followed by Alexa Fluor 647 anti-rabbit IgG and counterstained with Hoechst 33258 (Dojindo, Kumamoto, Japan). Resultant sections were examined by using a confocal laser microscope (Fluoview FV10i, Olympus, Tokyo, Japan) or a fluorescence microscope (Biozero, Keyence, Osaka, Japan).

### Quantitative Real-time RT-PCR

Total RNA was prepared from individual scaffolds using an RNeasy Mini Kit (Qiagen, Hilden, Germany), and cDNA was prepared using oligo(dT) primer and SuperScript III RT (Thermo Fisher Scientific). Real-time quantitative PCR was performed using SYBR Premix Ex Taq II and a Thermal Cycler Dice real-time system according to the manufacturer’s instructions (Takara, Otsu, Shiga, Japan). *Hypoxanthine phosphoribosyltransferase* (*HPRT*) was used as a housekeeping gene to normalize mRNA. Relative expression of real-time PCR products was determined by using the ΔΔCt method to compare target gene and *HPRT* mRNA expression. Primers used in this study are listed in Table S2 in Supplementary Material.

### Statistical Analysis

Data are presented as mean ± SD in triplicate. Statistical analyses were performed by using two-tailed Student’s *t-*test for comparisons of two groups and one-way analysis of variance and the Tukey–Kramer multiple comparison test for comparing more than three groups using GraphPad Prism 7 (GraphPad Software Inc., La Jolla, CA, USA). The receiver-operating characteristic curve and the area under the curve were used to assess the cut-off level between two groups. *P* < 0.05 was considered to indicate a statistically significant difference.

## Results

### Establishment of the 3D Coculture System

**Figure 2:**
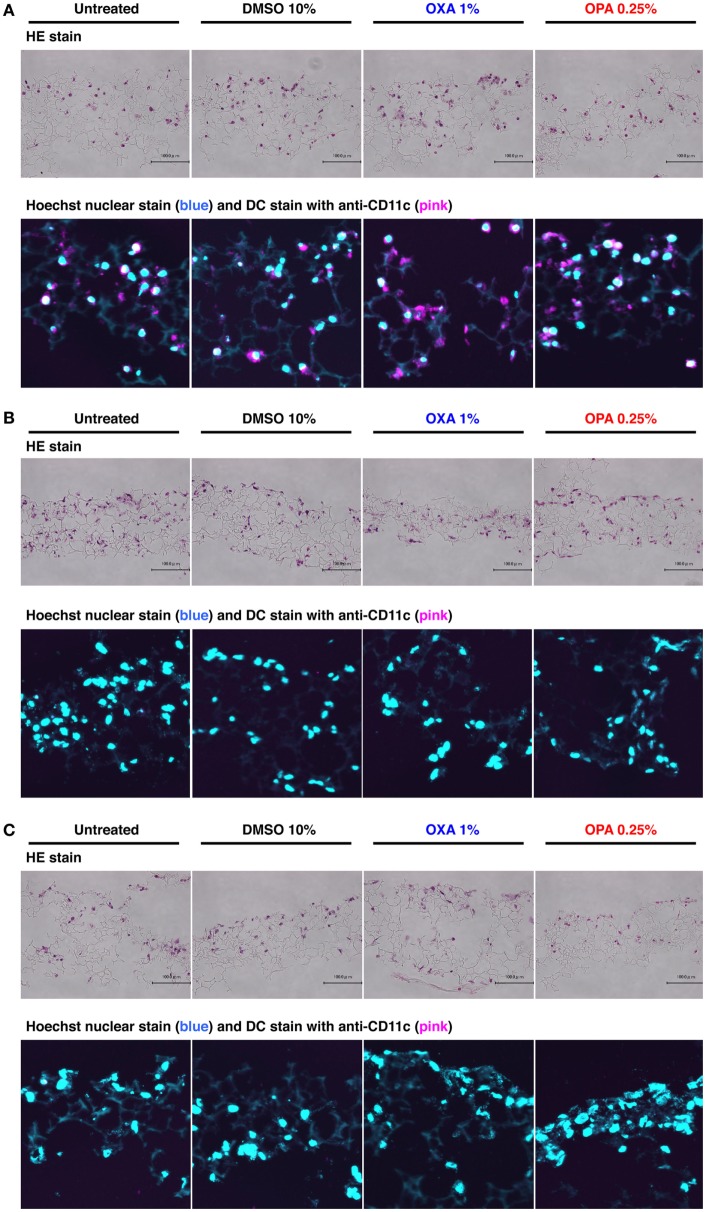
Chemical sensitizers failed to induce migration of dendritic cells (DCs) in the 3D coculture system. Typical skin and respiratory chemical sensitizers, oxazolone (OXA) and ortho-phthaldialdehyde (OPA), were added on the 3D coculture system. After stimulation for 24 h, immunohistochemical analysis in individual scaffolds of DC **(A)**, BEAS-2B **(B)**, and MRC-5 **(C)** was performed using anti-CD11c monoclonal antibody and nuclear staining with Hoechst together with hematoxylin and eosin (HE) staining to evaluate the migration of DCs. Representative confocal images of CD11c with Hoechst and HE staining are shown. Similar results were obtained in two independent experiments.

To mimic human upper airway epithelium, we first established a 3D coculture system consisting of airway epithelial cell line BEAS-2B, peripheral blood mononuclear cell-derived immature DCs, and lung fibroblast cell line MRC-5 (Figure 1), using the Alvetex scaffold, which is made of 200-μm-thick polystyrene and is porous with approximately 36–40 µm voids (23). The scaffold in the bottom of the insert well can be detached easily. In addition, the detached scaffolds can be attached sterilely to the bottom of the insert even after piling up, which is the best way to use this scaffold in this system. Histochemical analysis with HE staining revealed that the three sets of cells migrated into the respective scaffold and spread throughout it (Figure 2).

### Chemical Sensitizers Failed to Induce Migration of DCs into Other Scaffolds

A previous study showed that DCs migrate into the upper epithelial scaffold in response to challenge by a protein allergen (12), but the physiological relevance and molecular mechanism of this phenomenon remain elusive. After stimulation with OXA or OPA for 24 h, immunohistochemical analysis was performed using anti-CD11c mAb together with nuclear staining with Hoechst to evaluate the migration of DCs. In the scaffold layer of DCs, no obvious decrease in the number of DCs was observed after stimulation with either chemical sensitizer (Figure 2A). Consistent with this, almost no DCs were detected in the BEAS-2B and MRC-5 scaffolds (Figures 2B,C). When higher concentrations of sensitizers were used, although the number of DCs in the scaffold decreased, no migration of DCs into other scaffolds was observed (data not shown). Likewise, no migration of DCs into other scaffolds was observed after stimulation with DNCB or TMA, although some cytotoxicity was seen under these conditions (Figure S1 in Supplementary Material). Thus, in our 3D coculture system, DCs did not migrate into other scaffolds containing BEAS-2B or MRC-5 cells.

### OXA and OPA Similarly Upregulate CD86 but OPA Preferentially Upregulates OX40L in DCs

After disassembling the 3D coculture system, we performed mRNA expression analysis on individual scaffolds. We first evaluated the cytotoxicity of chemical sensitizers by analyzing the expression level of *HPRT*. The skin and respiratory chemical sensitizers, OXA and OPA, respectively, were added on the 3D coculture system. After stimulation for 3, 6, 9, and 12 h, RNA was extracted from individual scaffolds and subjected to real-time RT-PCR analysis. A dose-dependent decrease of mRNA expression of *HPRT* was observed in the DC scaffold after stimulation for 9 h (Figure S2 in Supplementary Material) and at other times (data not shown). A higher concentration of sensitizers greatly decreased the mRNA expression of *HPRT*, which was probably caused by degradation of RNA due to cell damage. Therefore, we did not use samples whose *HPRT* mRNA expression level was less than 1/10 that of untreated sample.

**Figure 3:**
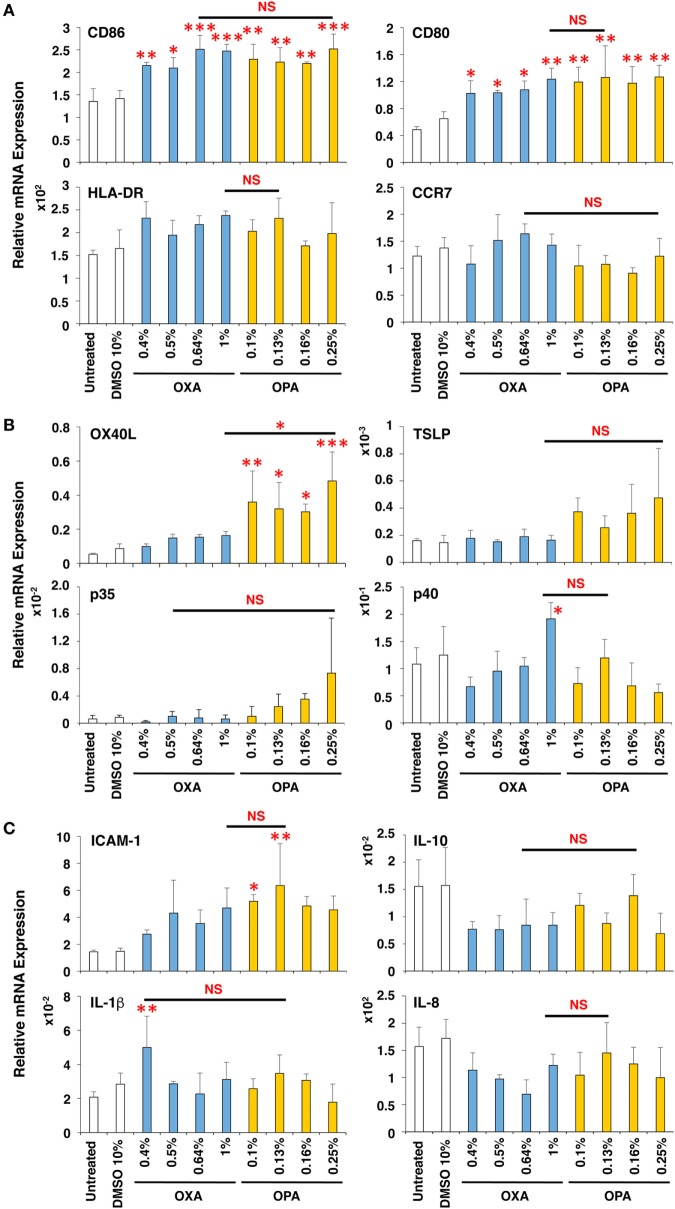
Oxazolone (OXA) and ortho-phthaldialdehyde (OPA) similarly upregulate CD86 but OPA preferentially upregulates OX40L in dendritic cells (DCs). Typical skin and respiratory chemical sensitizers, OXA and OPA, were added on the 3D coculture system. After stimulation for 9 h, RNA was extracted from the DC scaffold and subjected to real-time RT-PCR analysis to evaluate the expression of CD86, CD80, HLA-DR, CCR7 **(A)**, OX40L, thymic stromal lymphopoietin, interleukin (IL)-12p35, IL-12p40 **(B)**, intracellular adhesion molecule-1 (ICAM-1), IL-10, IL-1β, and IL-8 **(C)**. Data are shown as mean ± SD (*n* = 3) and are representative of more than three independent experiments. *P < 0.05; **P < 0.015; ***P < 0.001; NS, not significant.

The induction level of molecules important for DC maturation, such as co-stimulatory molecules CD86 and CD80, major histocompatibility antigen class II HLA-DR, and chemokine receptor CCR7, was examined after stimulation for 9 h (Figure 3A), which appeared to be the optimal time (data not shown). Both chemical sensitizers increased the expression of the co-stimulatory molecules, but not HLA-DR and CCR7, and no difference in the induction level was observed between chemical skin and respiratory sensitizers. In contrast, the expression of OX40L (also known as tumor necrosis factor ligand superfamily member 4, TNFSF4) was preferentially increased by stimulation with OPA but not with OXA (Figure 3B). This is noteworthy because OX40L was demonstrated to play a critical role in induction of Th2 differentiation through interaction with OX40 expressed on activated T cells (24, 25). However, thymic stromal lymphopoietin (TSLP), IL-12p35, IL-12p40, intracellular adhesion molecule-1 (ICAM-1), IL-10, IL-1β, and IL-8 did not show any significant and preferential upregulation by treatment with these chemical sensitizers (Figures 3B,C).

Thus, OXA and OPA similarly upregulate CD86 concomitant with DC maturation. However, during the DC maturation, OPA (but not OXA) preferentially augments the expression of OX40L.

### No Preferential Expression Was Observed in BEAS-2B and MRC-5 Cells by Stimulation with OXA and OPA

**Figure 4:**
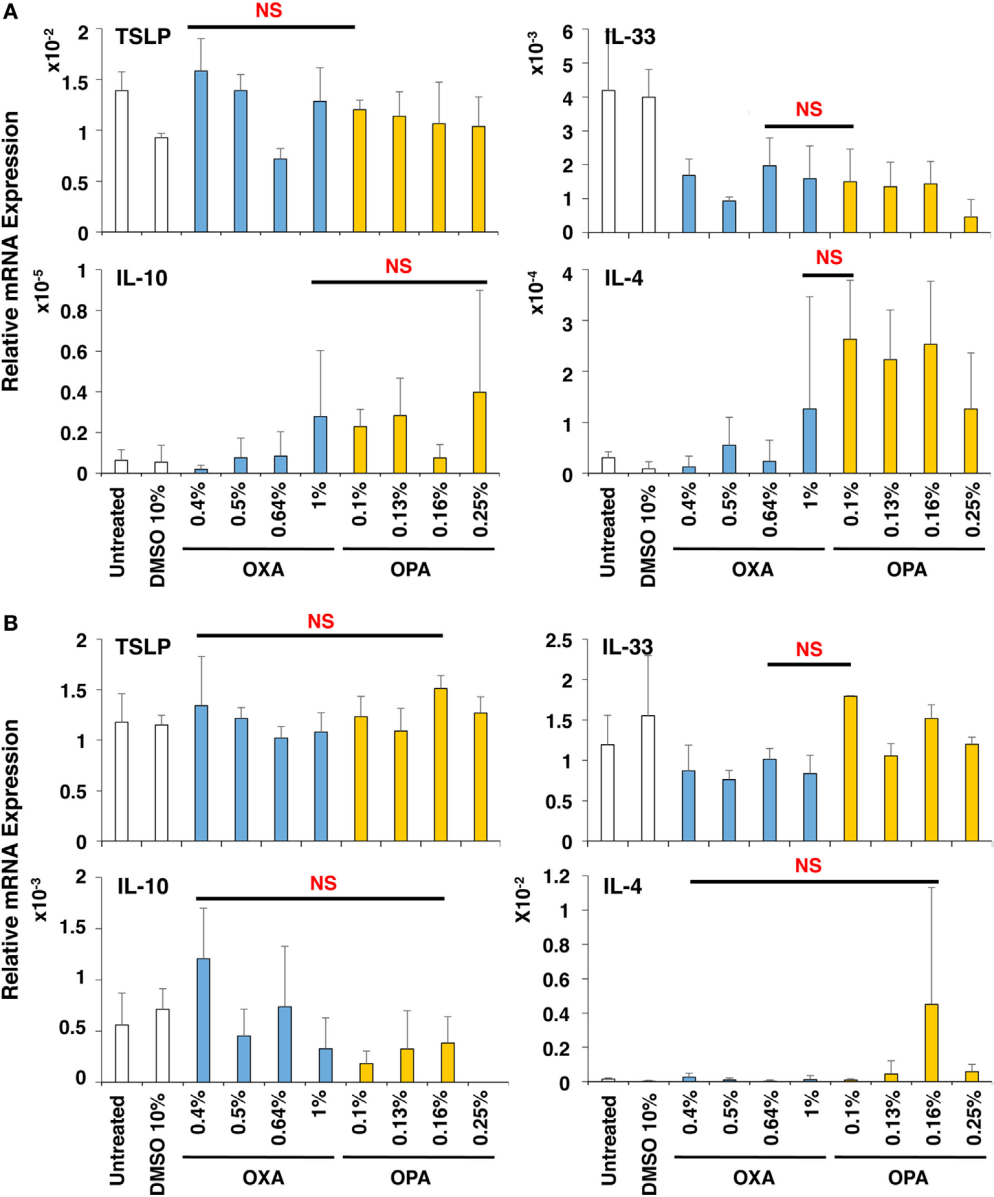
No preferential expression was observed in BEAS-2B and MRC-5 cells by stimulation with oxazolone (OXA) or ortho-phthaldialdehyde (OPA). Typical skin and respiratory chemical sensitizers, OXA and OPA, were added on the 3D coculture system. After stimulation for 9 h, RNA was extracted from the BEAS-2B **(A)** or MRC-5 **(B)** scaffold and subjected to real-time RT-PCR analysis to evaluate the expression of thymic stromal lymphopoietin, interleukin (IL)-33, IL-10, and IL-4. Data are shown as mean ± SD (*n* = 3) and are representative of more than three independent experiments. NS, not significant.

Next, the effects of chemical sensitizers on other molecules in upper airway epithelial cell line BEAS-2B and lung fibroblast cell line MRC-5 were examined. After stimulation with OXA or OPA for 9 h, RNA was extracted from the respective scaffolds and subjected to real-time RT-PCR analysis. However, we observed no significant and preferential upregulation of molecules including TSLP, IL-33, IL-10, and IL-4 (Figure 4), which were reported to be critically involved in the induction of Th2 differentiation and exertion of Th2 immune responses (20, 26, 27). Thus, no preferential expression was observed by stimulation with OXA or OPA in BEAS-2B and MRC-5 cells.

### FA and HDI Similarly Upregulate CD86 but HDI Preferentially Upregulates OX40L in DCs

**Figure 5:**
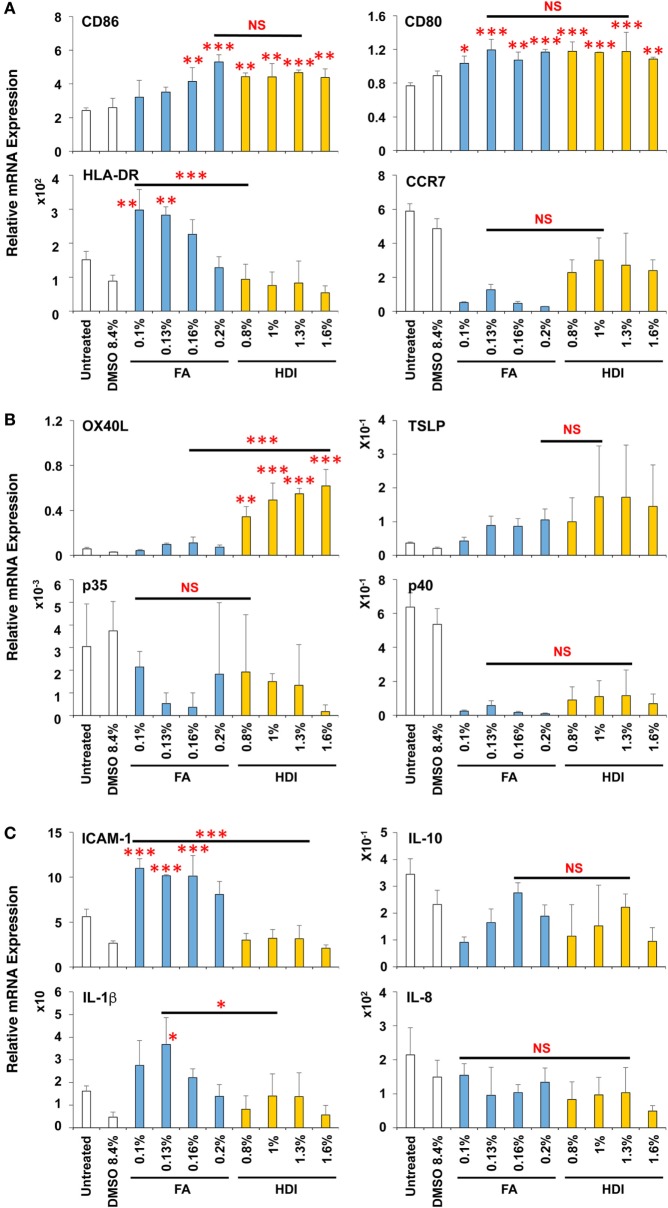
Formaldehyde (FA) and hexamethylene-1,6-diisocyanate (HDI) similarly upregulate CD86 but HDI preferentially upregulates OX40L in dendritic cells. Typical skin and respiratory chemical sensitizers, FA and HDI, were added on the 3D coculture system. After stimulation for 9 h, RNA was extracted from the DC scaffold and subjected to real-time RT-PCR analysis to evaluate the expression of CD86, CD80, HLA-DR, CCR7 **(A)**, OX40L, thymic stromal lymphopoietin, interleukin (IL)-12p35, IL-12p40 **(B)**, intracellular adhesion molecule-1 (ICAM-1), IL-10, IL-1β, and IL-8 **(C)**. Data are shown as mean ± SD (*n* = 3) and are representative of two independent experiments. *P < 0.05; **P < 0.015; ***P < 0.001; NS, not significant.

**Figure 6:**
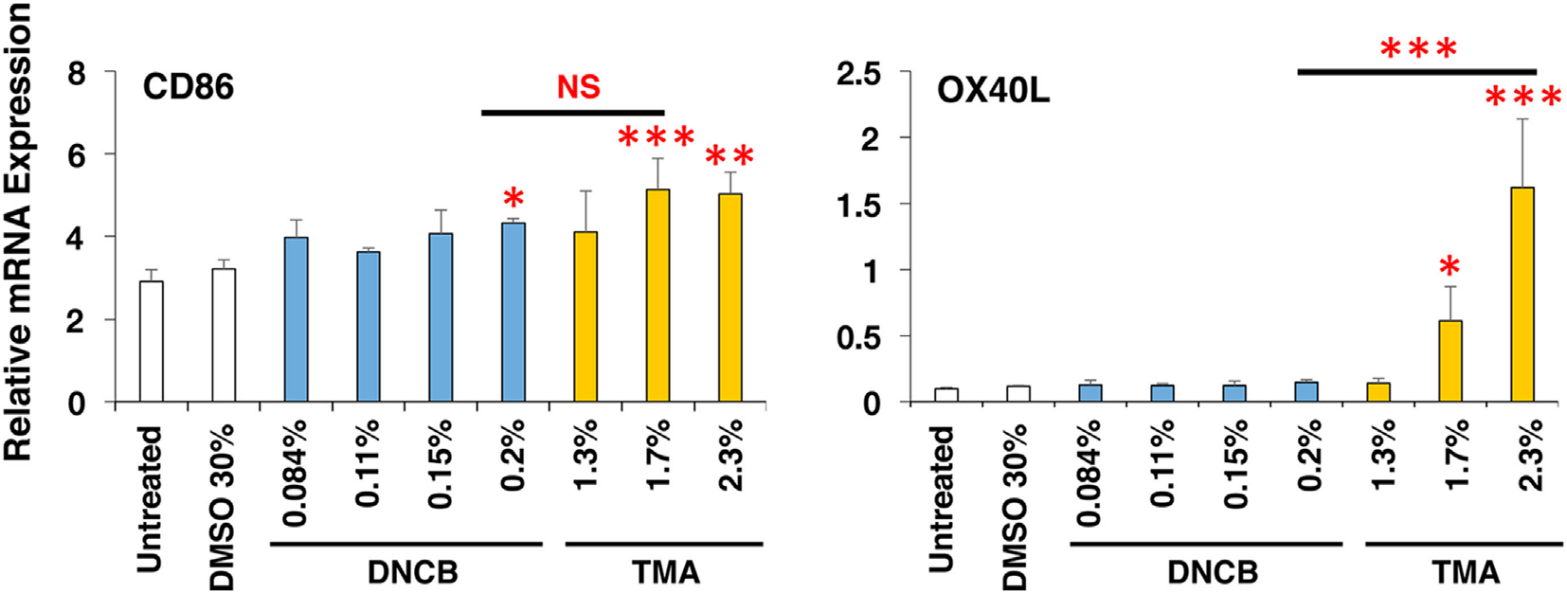
Dinitrochlorobenzene (DNCB) and trimellitic anhydride (TMA) similarly upregulate CD86 but TMA preferentially upregulates OX40L in dendritic cells. Typical chemical skin and respiratory sensitizers, DNCB and TMA, were added on the 3D coculture system. After stimulation for 9 h, RNA was extracted from the DC scaffold and subjected to real-time RT-PCR analysis to evaluate the expression of CD86 and OX40L. Data are shown as mean ± SD (*n* = 3) and are representative of more than three independent experiments. *P < 0.05; **P < 0.015; ***P < 0.001; NS, not significant.

To expand the applicability of this 3D coculture system to discriminate chemical skin and respiratory sensitizers, we used another set of typical skin and respiratory sensitizers, FA and HDI, respectively. FA and HDI both upregulate CD86 in DCs, and no difference was observed in the induction level of CD86 in DCs, suggesting similar induction of DC maturation (Figure 5A). Consistent with the results obtained by using OXA and OPA, HDI but not FA preferentially upregulated OX40L in DCs. Moreover, preferential upregulation of ICAM-1 and IL-1β was also observed by stimulation with FA as compared to that with HDI (Figures 5B,C). ICAM-1 was reported to be involved in the promotion of Th1 differentiation in T cells (28). No other preferential expression was observed by stimulation with them except for the preferential upregulation of IL-4 by FA in MRC-5 cells, which is opposite to what is expected (Figure S3 in Supplementary Material). Similar preferential upregulation of OX40L but comparable upregulation of CD86 in DCs was observed when the third set of chemical sensitizer, DNCB and TMA, was used (Figure 6). Collectively, these results suggest that both chemical skin and respiratory sensitizers similarly upregulate CD86 and induce DC maturation, but only respiratory sensitizers preferentially and consistently upregulate OX40L in DCs, indicating that OX40L expression in DCs is a good marker for discrimination between chemical respiratory and skin sensitizers.

### The 3D Coculture System Predicts Chemical Skin and Respiratory Sensitizers

**Figure 7:**
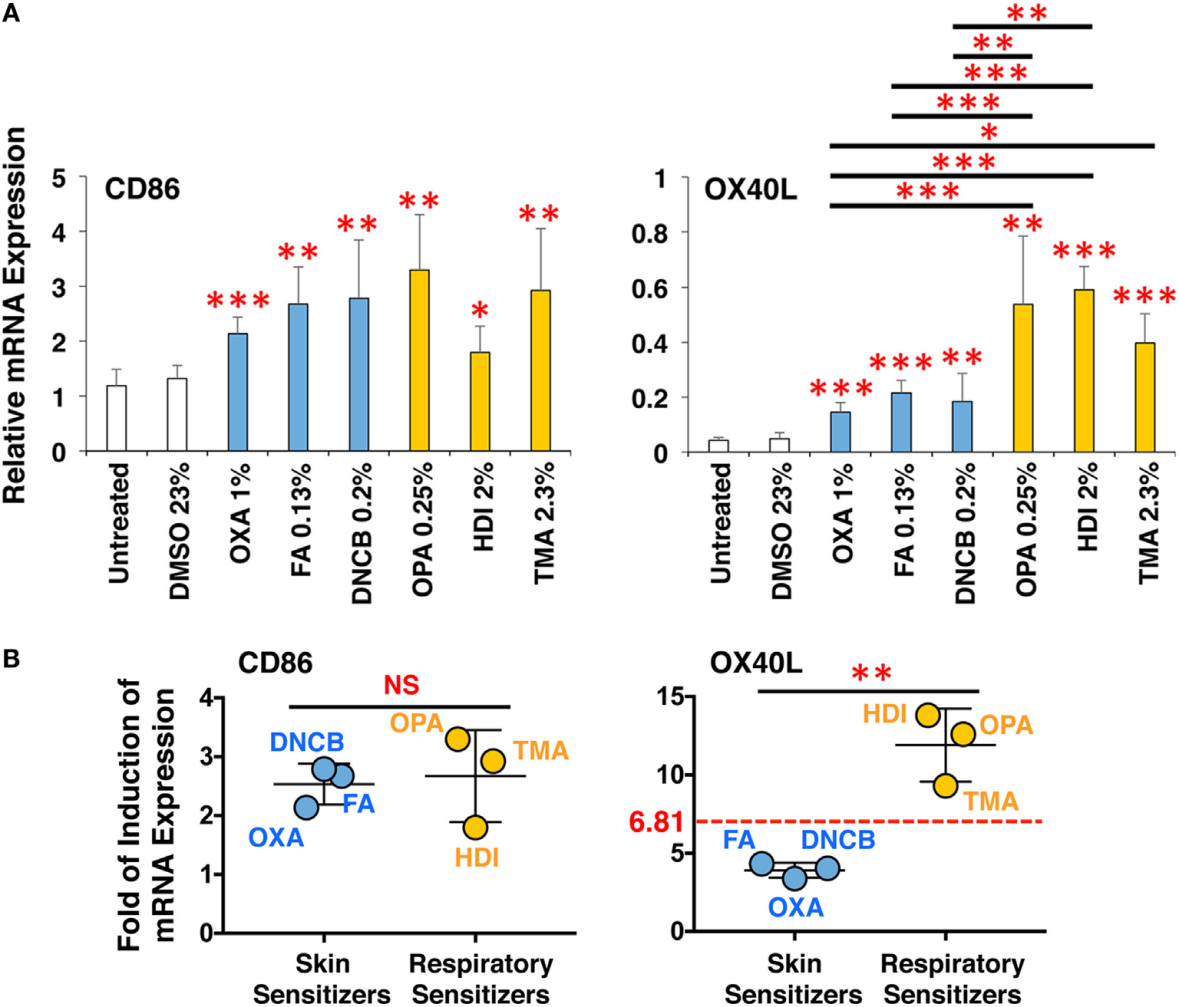
The 3D coculture system predicts chemical skin and respiratory sensitizers. All three sets of skin and respiratory chemical sensitizers, oxazolone (OXA), dinitrochlorobenzene (DNCB), and formaldehyde (FA) versus ortho-phthaldialdehyde (OPA), trimellitic anhydride (TMA), and hexamethylene-1,6-diisocyanate (HDI) were added on the 3D coculture system. After stimulation for 9 h, RNA was extracted from the DC scaffold and subjected to real-time RT-PCR analysis to evaluate the expression of CD86 and OX40L **(A)**. Fold of induction of CD86 and OX40L expression of all three respiratory sensitizers relative to untreated expression was calculated from the real-time RT-PCR data, and the mean-fold was compared between skin and respiratory sensitizers **(B)**. The cut-off level to discriminate skin and respiratory sensitizers was determined to be 6.81-fold by receiver-operating characteristic curve analysis. Data are shown as mean ± SD (*n* = 3) and are representative of three independent experiments. *P < 0.05; **P < 0.015; ***P < 0.001; NS, not significant.

Finally, to validate the applicability of this 3D coculture system to predict chemical skin and respiratory sensitizers, the responsiveness of all six chemical sensitizers was examined simultaneously using respective optimum concentrations. The sensitizers similarly upregulated CD86 in DCs and induced DC maturation (Figure 7A), but only the three respiratory sensitizers tended to more strongly upregulate OX40L. Moreover, an average of mean-fold of the induction of CD86 expression by sensitizers relative to the untreated expression was comparable between the sets of skin and respiratory sensitizers (Figure 7B). However, the average of mean-fold of the induction of OX40L expression of the respiratory sensitizers relative to untreated expression was significantly increased compared with that of the skin sensitizers; receiver-operating characteristic curve analysis revealed that the cut-off level to discriminate skin and respiratory sensitizers is 6.81-fold (Figure 7B).

## Discussion

A respiratory allergy such as asthma is predominantly caused by Th2 inflammatory responses in the airway, which are characterized by the induction of Th2 cytokines including IL-4, IL-5, and IL-13 that lead to IgE production, increased mucus production, and eosinophilia (11). This is in marked contrast to mixed Th immune responses—but mainly oriented toward Th1 responses—in contact dermatitis. Therefore, if one sensitizer can induce Th2 immune responses, it could potentially cause both respiratory and skin sensitization depending on where it elicits like HDI (19, 29). The main effector mechanism that induces Th2 polarization is the recognition of an allergen presented by DCs in local lymph nodes to naive CD4^+^ T cells. The differentiation of naive CD4^+^ T cells strongly depends on various co-stimulatory molecules expressed on the surface of T cells and their cognate ligands expressed on DCs and cytokines. One of the most critical co-stimulatory molecules is OX40 and its ligand, OX40L (24). OX40L upregulation is induced by TSLP, which is produced by epithelial cells, mast cells, and DCs (25). Moreover, a group of immune cells, the innate lymphoid cells, has recently been discovered (30). Among them, the Type-2 innate lymphoid cells produce Th2 cytokines upon activation by epithelial cell-derived cytokines such as IL-33 and IL-25, resulting in the promotion of inflammatory immune responses in the airways (27).

In the present study, the expression of these molecules in DCs, BEAS-2 cells, and MRC-5 cells was examined after stimulation with various chemicals. Among the molecules involved in Th2 differentiation and immune responses, only OX40L expression was significantly, consistently, and reproducibly upregulated by stimulation with typical respiratory sensitizers as compared to that with skin sensitizers. These results suggest that OX40L upregulation in DCs is the best marker for discriminating the chemical sensitization potential between skin and respiratory sensitizers.

To further confirm the advantage to use the 3D coculture system, we compared the expression of CD86 and OX40L in DCs between the 3D DC coculture system and DC monolayer system using OXA and OPA. The DC coculture system again showed preferential upregulation of OX40L by OPA but comparable upregulation of CD86 by OXA and OPA (Figure S4A in Supplementary Material). However, the DC monolayer system failed to preferentially upregulate the expression of OX40L by OPA with comparable upregulation of CD86 by OXA and OPA (Figure S4B in Supplementary Material). There results clearly indicate that the DC coculture system is superior to the DC monolayer system. Of note, in the case of DC monolayer system, we had to reduce the amounts of chemical sensitizers to apply on the system due to relatively higher cytotoxicity. This is presumably caused by more direct cytotoxic effect of chemical sensitizers on DCs, suggesting that the epithelial BEAS-2B cells in the coculture system function as a barrier to protect from external stimuli as in the case of *in vivo* physiological situation. In addition, for instance, the expression of TSLP, which was reported to induce OX40L upregulation (25), in DCs as well as in epithelial BEAS-2B cells and fibroblast MRC-5 cells has a tendency to more increase in response to respiratory sensitizers, although it is not statistically significant (Figures 3B and 5B; Figures S3A,B in Supplementary Material). Therefore, the cell–cell interactions among them also seem to contribute to the preferential upregulation of OX40L.

In the 3D coculture system described here, primary immature DCs, which were prepared from fresh human peripheral blood CD14^+^ monocytes each time, were used as the DC source. Actually, although we used blood collected from three healthy volunteers, we have obtained similar results by using these distinct volunteers’ blood (data not shown). However, to improve the system’s versatility, in place of primary DCs, we are currently trying to use iPS cell-derived DCs based on a protocol described previously (31, 32). In this case, we can use iPS cells prepared from patients suffering from allergies such as asthma, which might be of benefit to generate DCs highly sensitive to the chemical sensitization. Once we can select such useful iPS cells, they could be a good source to produce ideal DCs to discriminate skin and respiratory sensitizers. As a DC-like cell line, human acute myeloid leukemia cell line MUTZ-3 has been used after being differentiated into DCs by stimulation with GM-CSF and IL-4 (33). Using this cell line, Johansson and colleagues developed a genome-wide transcriptional profiling test platform, denoted the genomic allergen rapid detection assay (34). Using this assay, skin and respiratory sensitizers were successfully classified by their distinct biomarker signatures (35, 36). However, the biomarker signature for respiratory sensitizers does not seem to include any molecules known to be critically important for Th2 differentiation in DCs, such as OX40L (36). The authors explained this by noting that activation of DCs is a common phenomenon for both skin and respiratory sensitization (36). Research has shown, however, that there are several molecules critically important for Th2 differentiation in DCs leading to the development of asthma, and OX40L is one of them (24, 37, 38). Although it is easy to grow and maintain MUTZ-3 cells using standardized protocols, this difference might be attributed to limitations of using the MUTZ-3 cell line as a DC source.

A previous study demonstrated that DCs migrate into upper epithelial scaffold in response to challenge by a protein allergen using a similar but distinct 3D coculture system (12). Although the authors did not discuss the physiological relevance and molecular mechanism of this migration, no such migration was observed in our 3D coculture system (Figure 2; Figure S1 in Supplementary Material). The main reason for this different mobility is likely to be the distinct culture conditions, including the scaffold, epithelial cell line, allergen, and incubation time. However, the inability of DCs to migrate across scaffolds in our system is beneficial when analyzing mRNA expression in the respective cells after disassembling the system into the three scaffolds.

In the present study, a novel 3D coculture system consisting of upper airway epithelial cells, immature DCs, and lung fibroblast cells cultured in individual scaffolds was established. This system mimics the human airway upper epithelium and may be successfully applied to discriminate chemical respiratory sensitizers from skin sensitizers by measuring the critical molecule for Th2 differentiation, OX40L, in DCs. However, this system still has a limitation that only small sample numbers in each group are applied due to the limitation in the cell number to obtain primary immature DCs from a human donor. Although further verification of this assay system using more potential chemical sensitizers as well as improvements in the versatility are necessary, this 3D coculture system could be a good tool for evaluating the potential of chemical sensitization *in vitro*.

## Ethics Statement

This study was approved by the institutional review board of Tokyo Medical University (no. 3323). Written informed consent was obtained from all participants in accordance with the Declaration of Helsinki.

## Author Contributions

IM and TY designed the experiments and interpreted the results. IM, MO, YC, and HH performed the experiments and analyzed the data. IM and TY wrote the manuscript. MX and TO provided technical support and conceptual advice. TY supervised the project. All authors have read, discussed, and approved the final manuscript.

## Conflict of Interest Statement

The authors declare that the research was conducted in the absence of any commercial or financial relationships that could be construed as a potential conflict of interest.
