# Supplementary material for: Prediction of Chemical Respiratory and Contact Sensitizers by OX40L Expression in Dendritic Cells Using a Novel 3D Coculture System
Source: Front Immunol. 2017 Aug 4;8:929. doi: 10.3389/fimmu.2017.00929 (PMC5543289; doi:10.3389/fimmu.2017.00929)
Supplement: Supplementary file 1 [file Presentation_1.PDF]

SUPPLEMENTARY MATERIAL

Supplementary Table 1. Properties of chemical sensitizers used in this study.

Sensitizers	Catalog Number	Purity (%)	Molecular Weight	Specific Gravity	Concentration (mM) of 1% Solution
OXA	E0753	≥90	217.22		46.0
FA	F8775	36.5-38	30.03	1.09	333.0
DNCB	138630	97	202.55		49.4
OPA	P1378	≥97	134.13		74.5
HDI	52650	≥98	168.20	1.05	59.5
TMA	B4600	97	192.13		52.0

All chemical sensitizers used in this study were purchased form Sigma-Aldrich. The above information was obtained from individual data sheets of Sigma-Aldrich.

Supplementary Table 2. Primers used in this study.

Name	Direction	Sequence 5' to 3'
ICAM-1	forward	AACTGACACCTTTGTTAGCCACCTC
	reverse	TGTCCAGACATGACCGCTGA
CD80	forward	ATTATAAAGGCCAGCGCCAGAAC
	reverse	GGACAAATTCTACTTCCAGCAGCAC
CD86	forward	CTGTAACTCCAGCTCTGCTCCGTA
	reverse	GCCCATAAGTGTGCTCTGAAGTGA
HLA-DR	forward	TGACAAAGCGCTCCAACTATACTCC
	reverse	GAAGCCACGTGACATTGACCA
CCR7	forward	GGCCAACTTCAACATCACCAG
	reverse	GCCGATGAAGGCGTACAAGA
OX40L	forward	CAGTGCACATGCAGGCCTAAGTA
	reverse	GAAATATCCCTGTGTGGTTGCAGA
IL-1β	forward	TGAAGCAGCCATGGCAGAAG
	reverse	GGTCGGAGATTCGTAGCTGGA
IL-4	forward	CCGTAACAGACATCTTTGCTGCC
	reverse	GAGTGTCCTTCTCATGGTGGCT
IL-8	forward	TTCAGAGACAGCAGAGCACACA
	reverse	TTCACACAGAGCTGCAGAAATC
IL-10	forward	AACCTGCCTAACATGCTTCGAGA
	reverse	AACAACAAGTTGTCCAGCTGATCC
IL-12p35	forward	GAGGCCTGTTTACCATTGGAATTA
	reverse	TTCATGGTCTTGAACTCCACCTG
IL-12p40	forward	CTGGCCAGTACACCTGTCACAA
	reverse	CAGCAGGTGAAACGTCCAGAA
IL-33	forward	GCCTGTCAACAGCAGTCTACTG
	reverse	TGTGCTTAGAGAAGCAAGATACTC
TSLP	forward	TATCTGGTGCCCAGGCTATTCG
	reverse	TGAAGCGACGCCACAATCCTTG
HPRT	forward	GGCAGTATAATCCAAAGATGGTCAA
	reverse	GTCAAGGGCATATCCTACAACAAAC

Supplementary FIGURE 1. Chemical sensitizers failed to induce migration of DCs in the 3D co-culture system. Typical skin and respiratory chemical sensitizers, DNCB and TMA, were added on the 3D co-culture system. After stimulation for 24 h, immunohistochemical analysis of individual scaffolds of DC (A), BEAS-2B (B), and MRC-5 (C) was performed using anti-CD11c mAb and nuclear staining with Hoechst together with HE staining to evaluate the migration of DCs. Representative confocal images of CD11c with Hoechst and HE staining are shown. Similar results were obtained in more than three independent experiments.

Supplementary FIGURE 2. Evaluation of cytotoxicity of chemical sensitizers by *HPRT* mRNA expression level. Skin and respiratory chemical sensitizers, OXA and OPA, respectively, were added on the 3D co-culture system. After stimulation for 9 h, RNA was extracted from the DC scaffold and subjected to real-time RT-PCR analysis of *HPRT* expression. We decided not to use samples whose *HPRT* mRNA expression level was less than 1/10 (red column) that of untreated sample.

Supplementary FIGURE 3. No preferential expression was observed in BEAS-2B and MRC-5 cells by stimulation with FA and HDI. Typical skin and respiratory chemical sensitizers, FA and HDI, were added on the 3D co-culture system. After stimulation for 9 h, RNA was extracted from the BEAS-2B (A) and MRC-5 (B) scaffold and subjected to real-time RT-PCR analysis to evaluate the expression of TSLP, IL-33, IL-10, and IL-4. Data are shown as mean ± SD (n = 3) and are representative of two independent experiments. NS, not significant.

Supplementary FIGURE 4. 3D DC co-culture system is superior to DC monolayer system. Typical skin and respiratory chemical sensitizers, OXA and OPA, were added on the 3D DC co-culture system (A) and 3D DC monolayer system (B). After stimulation for 9 h, RNA was extracted from the DC scaffold and subjected to real-time RT-PCR analysis to evaluate the expression of CD86, and OX40L. Note that the percentages of chemical sensitizers added on the DC monolayer system are much less than those on the DC co-culture system due to relatively higher cytotoxicity. Data are shown as mean ± SD (n = 3) and are representative of two independent experiments. NS, not significant.

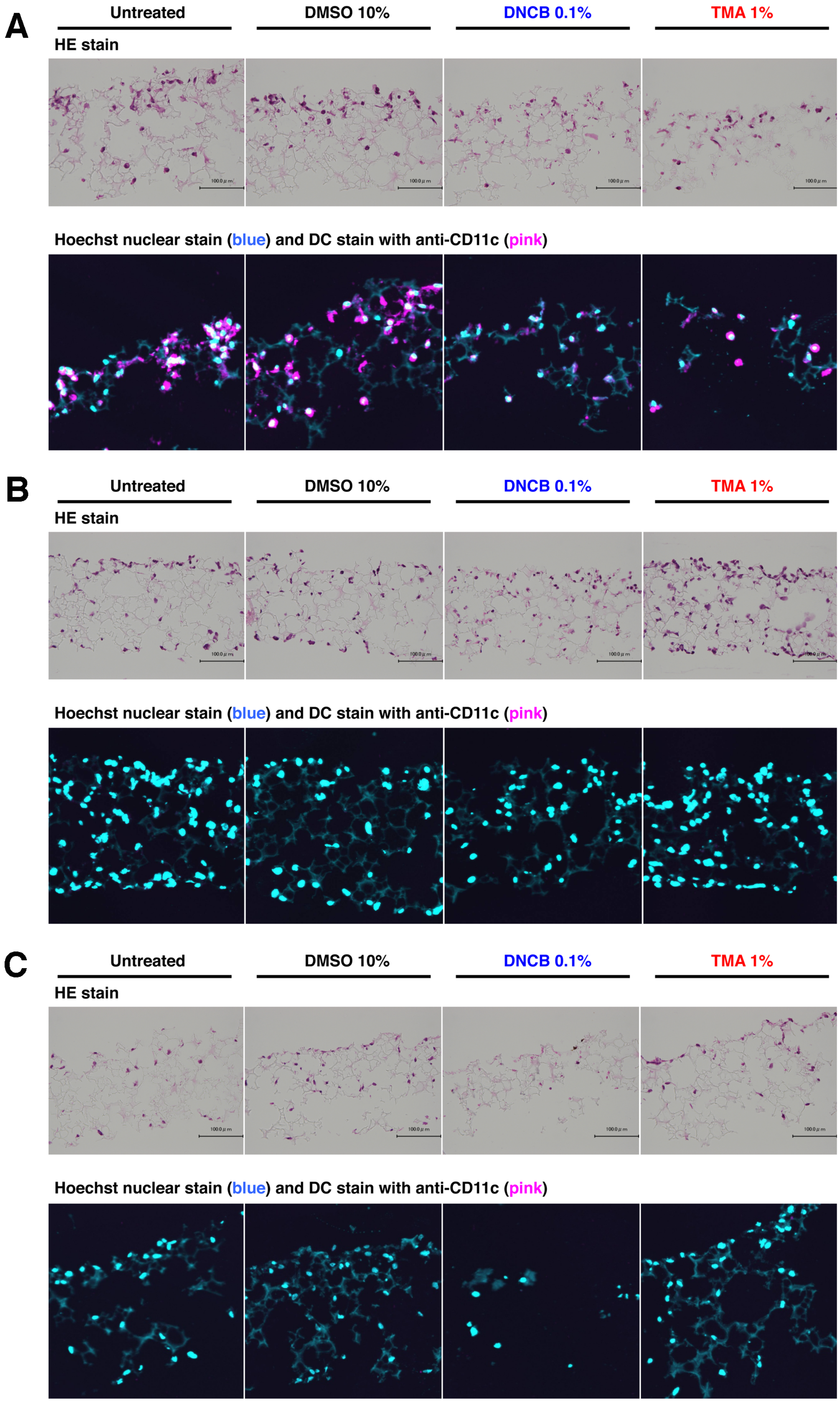

Supplementary Fig. 1. Mizoguchi et al.

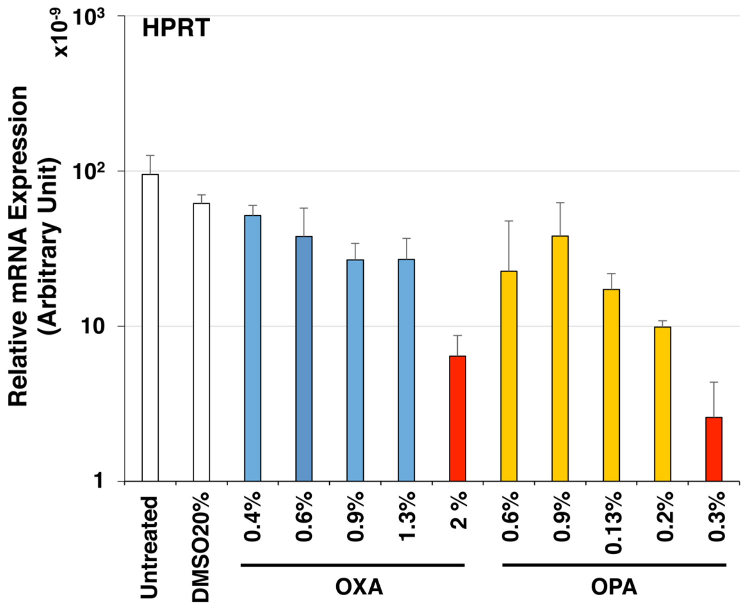

Supplementary Fig. 2. Mizoguchi et al.

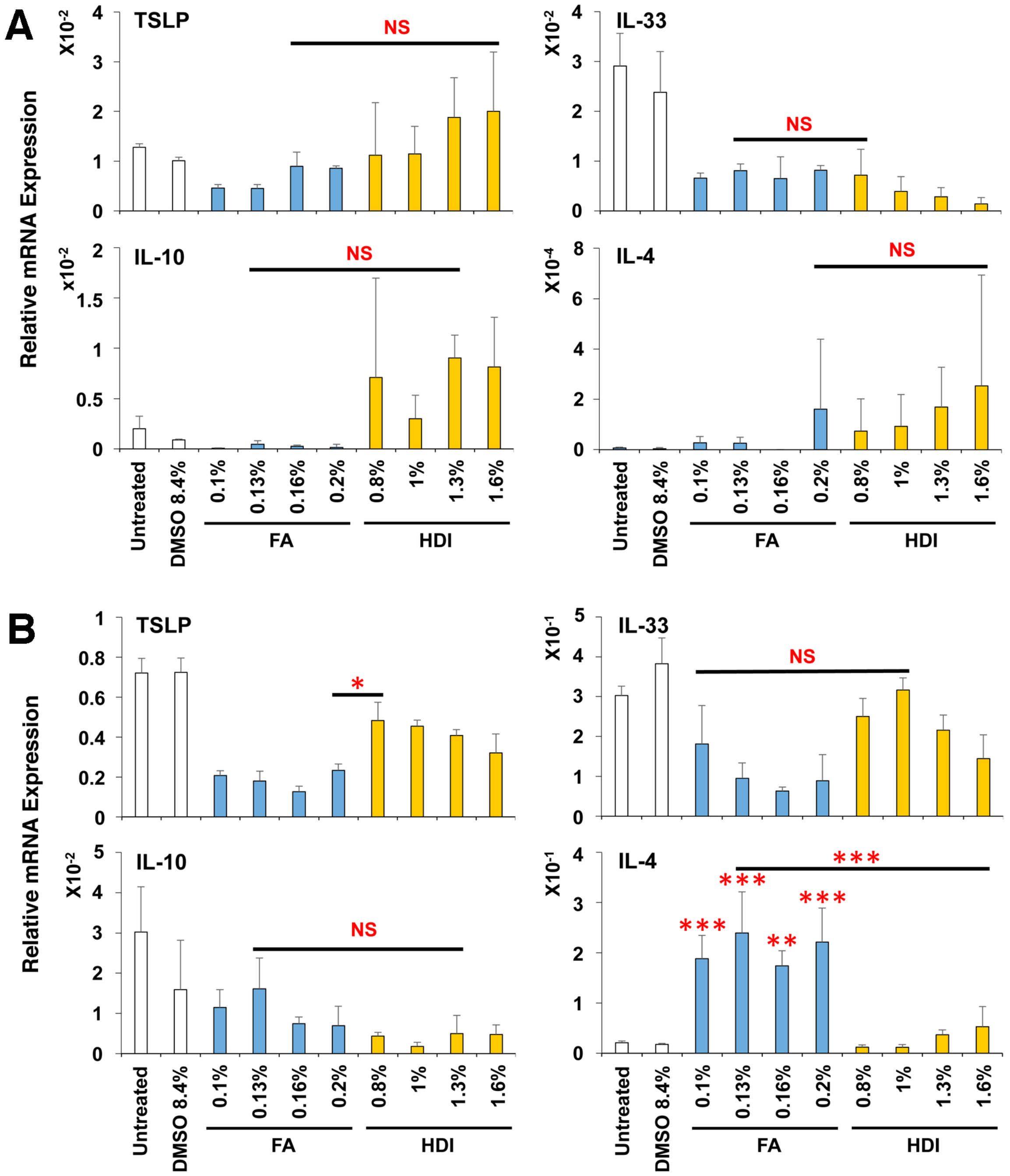

Supplementary Fig. 3. Mizoguchi et al.

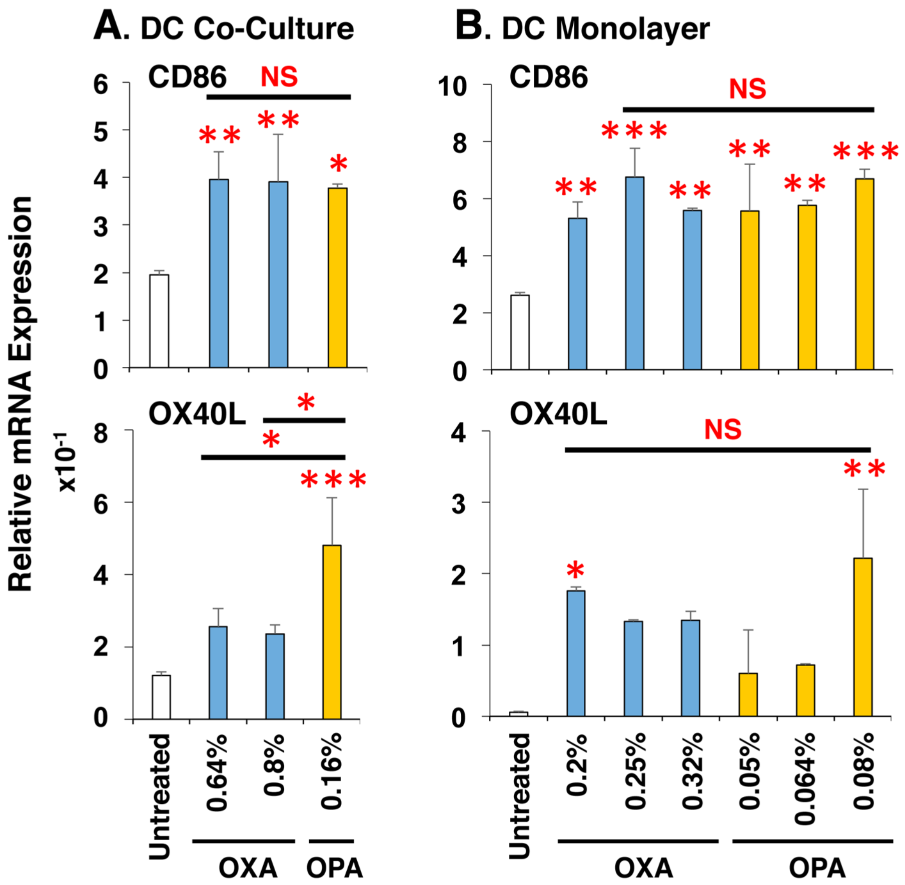

Supplementary Fig. 4. Mizoguchi et al.
